# Supplementary material for: Neural space–time model for dynamic multi-shot imaging
Source: Nat Methods. 2024 Sep 24;21(12):2336–41. doi: 10.1038/s41592-024-02417-0 (PMC11621023; doi:10.1038/s41592-024-02417-0)
Supplement: Supplementary file 1 — Supplementary Text, Figs. 1 and 2 and Table 1. [file 41592_2024_2417_MOESM1_ESM.pdf]

nature methods

Supplementary information https://doi.org/10.1038/s41592-024-02417-0

Neural space–time model for dynamic multi-shot imaging

In the format provided by the authors and unedited

Supplementary Text on Hash embedding

Hash embedding store all features vector in its weights, *i.e.,* a list of feature storage array, $[\psi_0, \psi_1, \cdots, \psi_{N-1}]$. The hash embedding transforms a coordinate vector to a multi-resolution feature vector, $\boldsymbol{h} = [h_0, h_1, \cdots, h_{N-1}]$. Starting from the basic case, when the coordinate vector (x) is *1*-D. To obtain the feature at a particular resolution h_i, we first identify the nearest coordinate values on the resolution grid that are just greater or smaller than x, which are denoted as $\lceil x \rceil$ and $\lfloor x \rfloor$. Then, we define a fixed hash function to obtain the hash values for $\lceil x \rceil$ and $\lfloor x \rfloor$, and the hash values are used to retrieve the features corresponding to $\lceil x \rceil$ and $\lfloor x \rfloor$ from the ψ_i. Lastly, we linearly interpolate these retrieved features for the feature for x. Putting this mathematically,

$$h_i\left(x\right) = \left(x - \lfloor x \rfloor\right) \cdot \psi\left(hash\left(\lceil x \rceil\right)\right) + \left(\lceil x \rceil - x\right) \cdot \psi\left(hash\left(\lfloor x \rfloor\right)\right). \tag{8}$$

Generalizing this into N-D, we will find 2^N nearest coordinate vectors and perform N-D interpolation based on 2^N of the retrieved features. By repeating this process for each resolution, we concatenate features from all resolutions for the hash embedded features as the input of the coordinate-based neural network. In our notation, the hash embedding weights, ψ, are considered as a part of the network weights, θ, and thus ψ is not written out in Eqs. 1-3. They are updated together using the same learning setting the during the reconstruction.

Imaging system, reconstruction dimension, number of epochs	1× NVIDIA RTX 3090 (24GB GPU RAM), Intel Xeon Gold 6226R	1× NVIDIA A6000 (48GB GPU RAM), Intel Xeon Gold 6444Y	1× NVIDIA A100 (80GB GPU RAM), Intel Xeon Gold 6144
DPC (Fig. 1c) 320×1000, 4 timepoints 5000 epochs	6.04 minutes	4.40 minutes	4.35 minutes
SIM (Fig. 2) 320×320, 15 timepoints 2000 epochs	3.64 minutes	2.71 minutes	3.70 minutes
3D SIM (Fig. 3) $20 \times 512 \times 512$, 15 timepoints 500 epochs	insufficient GPU RAM	40.5 minutes	43.2 minutes
Rolling-shutter DiffuserCam (Extended Data Fig. 7) 540×640, 280 timepoints 200 epochs	94.1 minutes	81.7 minutes	73.8 minutes

Supplementary Table 1 The runtime of NSTM reconstructions under different GPU models. The CPU model is also listed as a reference. All computations were performed using single-precision arithmetic and JAX library. While this serves as a reference for the processing speed of NSTM, the actual runtime also varies based on other computer configurations (*e.g.* NVIDIA driver and CUDA software versions, CPU and RAM speed, computer I/O speed, etc.).

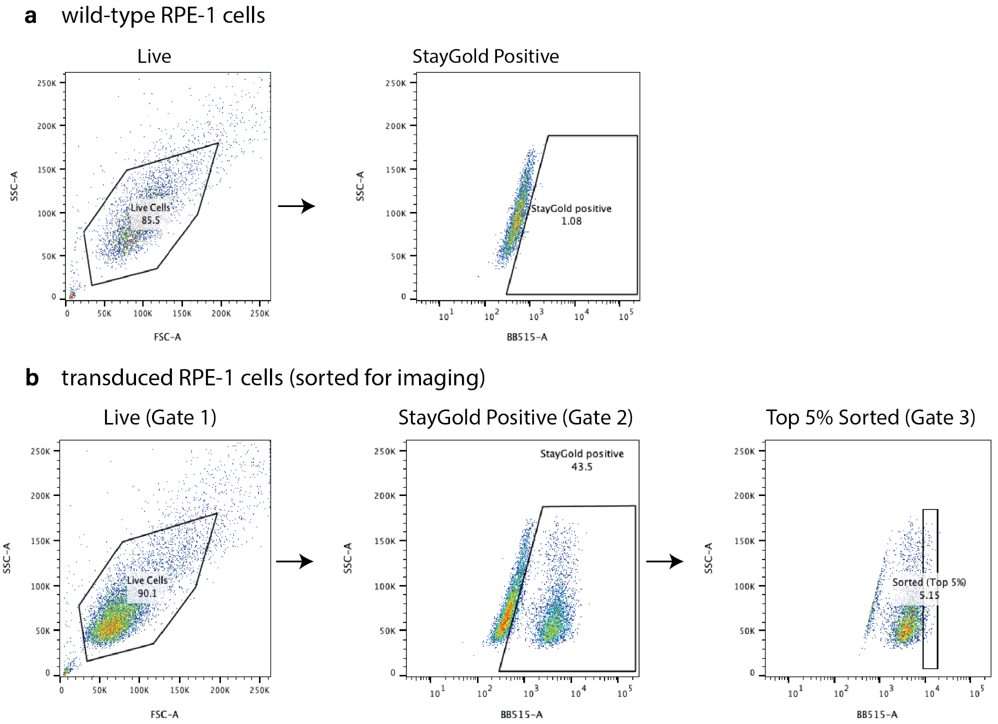

Supplementary Fig. 1 Gating strategy for sorting the StayGold tagged- ER and mitochondrial matrix lines. **a**, Wild-type RPE-1 cells were used to gate for Live Cells (Gate 1) and the StayGold negative cells were used to gate for the StayGold positive population (Gate 2). **b**, To sort samples that were transduced with StayGold expressing plasmids, Gate 1 (Live cells) was applied followed by Gate 2 (StayGold positive), and then top 5% of the StayGold positive cell population (Gate 3) was sorted using BDFACS Aria Fusion Sorter and expanded using DMEM-F12 for subsequent imaging experiments.

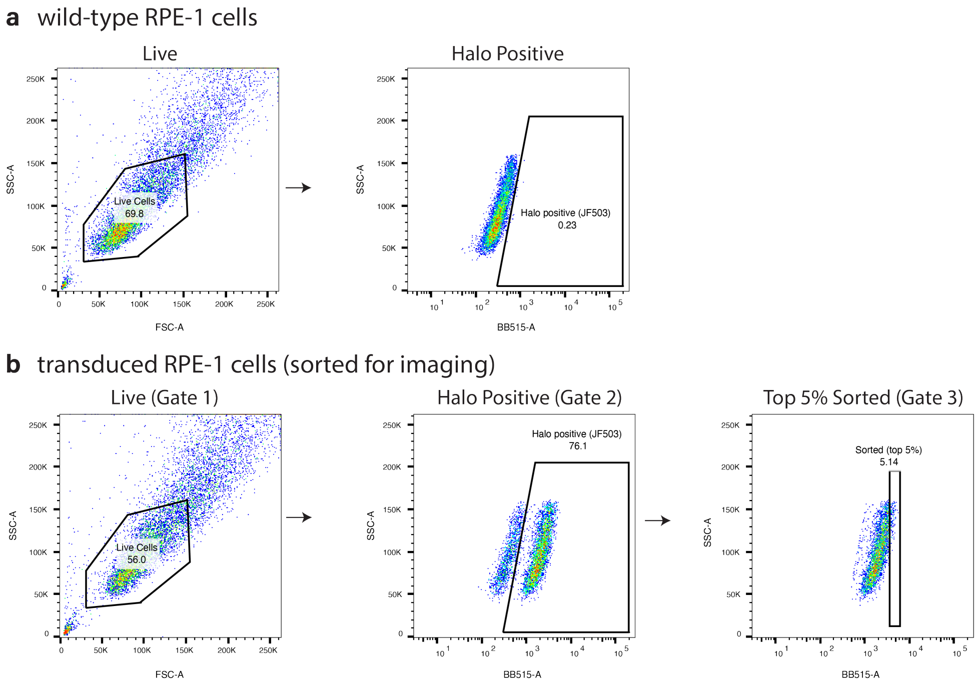

Supplementary Fig. 2 Gating strategy for sorting the LifeAct-Halo tagged RPE-1 line. **a**, Wild-type RPE-1 cells were used to gate for Live Cells (Gate 1) and the Halo negative cells were used to gate for the Halo positive population (Gate 2). **b**, To sort samples that were transduced with Halo expressing plasmids, Gate 1 (Live cells) was applied followed by Gate 2 (Halo positive), and then top 5% of the Halo positive cell population (Gate 3) was sorted using BDFACS Aria Fusion Sorter and expanded using DMEM-F12 for subsequent imaging experiments.
