# Supplementary material for: Neural space–time model for dynamic multi-shot imaging
Source: Nat Methods. 2024 Sep 24;21(12):2336–41. doi: 10.1038/s41592-024-02417-0 (PMC11621023; doi:10.1038/s41592-024-02417-0)
Supplement: Supplementary file 2 — Reporting Summary [file 41592_2024_2417_MOESM2_ESM.pdf]

nature portfolio

Corresponding author(s): Ruiming Cao, Laura Waller

Last updated by author(s): Aug 3, 2024

Reporting Summary

Nature Portfolio wishes to improve the reproducibility of the work that we publish. This form provides structure for consistency and transparency in reporting. For further information on Nature Portfolio policies, see our Editorial Policies and the Editorial Policy Checklist.

Statistics

For all statistical analyses, confirm that the following items are present in the figure legend, table legend, main text, or Methods section.

n/a	Confirmed	
☐	☒	The exact sample size (*n*) for each experimental group/condition, given as a discrete number and unit of measurement
☒	☐	A statement on whether measurements were taken from distinct samples or whether the same sample was measured repeatedly
☒	☐	The statistical test(s) used AND whether they are one- or two-sided<br>*Only common tests should be described solely by name; describe more complex techniques in the Methods section.*
☐	☒	A description of all covariates tested
☐	☒	A description of any assumptions or corrections, such as tests of normality and adjustment for multiple comparisons
☒	☐	A full description of the statistical parameters including central tendency (e.g. means) or other basic estimates (e.g. regression coefficient) AND variation (e.g. standard deviation) or associated estimates of uncertainty (e.g. confidence intervals)
☒	☐	For null hypothesis testing, the test statistic (e.g. *F*, *t*, *r*) with confidence intervals, effect sizes, degrees of freedom and *P* value noted<br>*Give P values as exact values whenever suitable.*
☒	☐	For Bayesian analysis, information on the choice of priors and Markov chain Monte Carlo settings
☒	☐	For hierarchical and complex designs, identification of the appropriate level for tests and full reporting of outcomes
☒	☐	Estimates of effect sizes (e.g. Cohen's *d*, Pearson's *r*), indicating how they were calculated

Our web collection on statistics for biologists contains articles on many of the points above.

Software and code

Policy information about availability of computer code

Data collection	Zeiss Zen Black (v14.0.9.201)
Data analysis	Our main method was implemented with Python 3.9, jax v0.3.18, flax v0.6.0, CalCIL v0.0.1 (https://github.com/rmcao/CalCIL), nstm v0.1 (https://github.com/rmcao/nstm). Baseline study: cuda-accelerated three-beam 3D SIM reconstruction software (cudasirecon) v1.2.0 (https://github.com/scopetools/cudasirecon), fairSIM v1.5.0 (https://www.fairsim.org). Visualizations were made with matplotlib v3.5.3, napari v0.4.18 (for Supplementary Video 3 and 5). Cell sorting was performed with BD FACSDiva v9.0.2 and FlowJo v10.10.0.

For manuscripts utilizing custom algorithms or software that are central to the research but not yet described in published literature, software must be made available to editors and reviewers. We strongly encourage code deposition in a community repository (e.g. GitHub). See the Nature Portfolio guidelines for submitting code & software for further information.

Data

Policy information about availability of data

All manuscripts must include a data availability statement. This statement should provide the following information, where applicable:

- Accession codes, unique identifiers, or web links for publicly available datasets
- A description of any restrictions on data availability
- For clinical datasets or third party data, please ensure that the statement adheres to our policy

Our code is available at https://github.com/rmcao/nstm. SIM datasets collected in this study were deposited in Zenodo at https://doi.org/10.5281/

zenodo.13204660. DPC and rolling-shutter DiffuserCam datasets were obtained from Biomed. Opt. Express 9, 5456-5466 (2018) and https://doi.org/10.1109/ICCPHOT.2019.8747341, and are also available at https://github.com/rmcao/nstm.

Human research participants

Policy information about studies involving human research participants and Sex and Gender in Research.

Reporting on sex and gender	n/a
Population characteristics	n/a
Recruitment	n/a
Ethics oversight	n/a

Note that full information on the approval of the study protocol must also be provided in the manuscript.

Field-specific reporting

Please select the one below that is the best fit for your research. If you are not sure, read the appropriate sections before making your selection.

☒ Life sciences ☐ Behavioural & social sciences ☐ Ecological, evolutionary & environmental sciences

For a reference copy of the document with all sections, see nature.com/documents/nr-reporting-summary-flat.pdf

Life sciences study design

All studies must disclose on these points even when the disclosure is negative.

Sample size	We chose a diverse set of imaging systems and samples to demonstrate our method. No sample size-based statistics is involved in this study, as this study focus on microscopy reconstruction method and does not have any hypothesis testing.
Data exclusions	No data was excluded.
Replication	The results of NSTM reconstruction can be replicated using the processing software included in the submission files.<br>The microbead with vibrating motion experiment shown in Fig.2 was independently performed nine times. The optical table was pushed and released in each time. Seven out of nine acquired datasets are suitable for NSTM reconstruction and produce similar results. The remaining two datasets suffer from severe motion blur in individual raw images and thus cannot be recovered by NSTM.
Randomization	Neural networks are initialized with random weights, and the dataset is randomly ordered during each epoch of training. Besides, randomization was not relevant to our study, as our study does not have any hypothesis testing.
Blinding	Blinding was not relevant to our study as our method was based on computational metrics and algorithms that do not require subjective assessment.

Reporting for specific materials, systems and methods

We require information from authors about some types of materials, experimental systems and methods used in many studies. Here, indicate whether each material, system or method listed is relevant to your study. If you are not sure if a list item applies to your research, read the appropriate section before selecting a response.

Materials & experimental systems

n/a	Involved in the study	
☒	☐	Antibodies
☐	☒	Eukaryotic cell lines
☒	☐	Palaeontology and archaeology
☒	☐	Animals and other organisms
☒	☐	Clinical data
☒	☐	Dual use research of concern

Methods

n/a	Involved in the study	
☒	☐	ChIP-seq
☐	☒	Flow cytometry
☒	☐	MRI-based neuroimaging

Eukaryotic cell lines

Policy information about cell lines and Sex and Gender in Research

Cell line source(s)	The cell line hTERT RPE-1 was obtained from ATCC (https://www.atcc.org/products/crl-4000); gender female. The HEK293t cell line was obtained from ATCC https://www.atcc.org/products/crl-3216; gender female.
Authentication	ATCC cell lines arrive with certificate of analysis and obtained from the UC Berkeley Biosciences Division Cell Culture Facility. These lines are distributed commercially.
Mycoplasma contamination	The cell lines were tested negative for mycoplasma.
Commonly misidentified lines (See ICLAC register)	The RPE-1 and HEK293t cells are not part of commonly misidentified lines.

Flow Cytometry

Plots

Confirm that:

☒ The axis labels state the marker and fluorochrome used (e.g. CD4-FITC).

☒ The axis scales are clearly visible. Include numbers along axes only for bottom left plot of group (a 'group' is an analysis of identical markers).

☒ All plots are contour plots with outliers or pseudocolor plots.

☒ A numerical value for number of cells or percentage (with statistics) is provided.

Methodology

Sample preparation	The RPE-1 cells were transformed using lentivirus transduction as described in the materials and methods, they were suspended using trypsin, and cultured using DMEM F-12 media. The cells were kept on ice at all times during sorting. After sorting they were spun down and resuspended and plated in 10 cm dishes before being used for imaging experiments.
Instrument	BD FACSAria (TM) Fusion Cell Sorter (HHMI) was used the in the LSA flow cytometry core at UC Berkeley.
Software	BD FACSDiva Software and FlowJo was used to collect and analyze flow cytometry data.
Cell population abundance	The cells were approximately 95% (ER) and 78% (mitochondria) StayGold positive respectively after sorting. The halo-tagged F-Actin cells were 99% halo positive after sorting.
Gating strategy	The StayGold negative cells were used for gating within live cells using wild-type RPE cells with no fluorophore. This gate was used to designate the StayGold positive cells. Within this population, approximately top 5% of StayGold positive cells were sorted for use in imaging experiments. Gating strategy included in the supplementary figure. The halo negative cells were used for gating within live cells using wild-type RPE cells with no fluorophore. This gate was used to designate the Halo positive cells in the BB515A channel (due to use of the JF503 ligand to gate Halo-positive cells). Within the Halo positive population, top 5% of cells were sorted. Gating strategy is included in supplementary figure.

☒ Tick this box to confirm that a figure exemplifying the gating strategy is provided in the Supplementary Information.
